# Transorbital point-of-care ultrasound versus fundoscopic papilledema to support treatment indication for potentially elevated intracranial pressure in children

**DOI:** 10.1007/s00381-023-06186-7

**Published:** 2023-11-14

**Authors:** Susanne Regina Kerscher, Julian Zipfel, Karin Haas-Lude, Andrea Bevot, Jonas Tellermann, Martin Ulrich Schuhmann

**Affiliations:** 1 Department of Diagnostic and Interventional Radiology, University Hospital Ulm, Albert-Einstein-Allee 23, Ulm, 89081 Germany; 2 Department of Neurosurgery, Division of Paediatric Neurosurgery, University Hospital of Tuebingen, Tübingen, Germany; 3 Department of Paediatric Neurology and Developmental Medicine, University Children’s Hospital of Tuebingen, Tübingen, Germany

**Keywords:** Fundoscopy, Optic nerve sheath diameter, US-based optic disc, Elevated ICP

## Abstract

**Purpose:**

To compare transorbital point-of-care ultrasound techniques —optic nerve sheath diameter (US-ONSD) and optic disc elevation (US-ODE)— with fundoscopic papilledema to detect potentially raised intracranial pressure (ICP) with treatment indication in children.

**Methods:**

In a prospective study, 72 symptomatic children were included, 50 with later proven disease associated with raised ICP (e.g. pseudotumour cerebri, brain tumour, hydrocephalus) and 22 with pathology excluded. Bilateral US-ONSD and US-ODE were quantified by US using a 12-MHz-linear-array transducer. This was compared to fundoscopic optic disc findings (existence of papilledema) and, in 28 cases, invasively measured ICP values.

**Results:**

The sensitivity and specificity of a cut-off value of US-ONSD (5.73 mm) to detect treatment indication for diseases associated with increased ICP was 92% and 86.4%, respectively, compared to US-ODE (0.43 mm) with sensitivity: 72%, specificity: 77.3%. Fundoscopic papilledema had a sensitivity of 46% and a specificity of 100% in this context. Repeatability and observer-reliability of US-ODE examination was eminent (Cronbach’s *α* = 0.978–0.989). Papilledema was detected fundoscopically only when US-ODE was > 0.67 mm; a US-ODE > 0.43 mm had a positive predictive value of 90% for potentially increased ICP.

**Conclusion:**

In our cohort, transorbital point-of-care US-ONSD and US-ODE detected potentially elevated ICP requiring treatment in children more reliably than fundoscopy. US-ONSD and US-ODE indicated the decrease in ICP after treatment earlier and more reliably than fundoscopy. The established cut-off values for US-ONSD and US-ODE and a newly developed US-based grading of ODE can be used as an ideal first-line screening tool to detect or exclude conditions with potentially elevated ICP in children.

## Introduction

Children often have symptoms reminiscent of increased intracranial pressure (ICP), e.g. nausea, vomiting or headaches. The exclusion of a disease associated with an increased ICP, e.g. brain tumours, hydrocephalus or pseudotumour cerebri (PTC), is therefore regularly necessary in paediatric medicine. Fundoscopy to detect/exclude papilledema is common first-line-practice if raised ICP is suspected [1]. However, correct fundoscopic diagnosis of papilledema is often challenging, especially for non-ophthalmologists [2–4], and even among trained practitioners [5–7]. Furthermore, in 40–60% of children with elevated ICP, papilledema can be absent [8–13].

Point-of-care (PoC) ultrasound of the optic nerve sheath diameter (US-ONSD) provides an alternative non-invasive, rapid and reliable method to estimate ICP in children [14–16], demonstrating a good correlation to invasively measured ICP [17–19] and a high intra- and inter-observer reliability [15]. Transorbital ultrasound can also be used to detect an elevation of the optic disc (US-ODE) [20], and this technique has been described as a useful adjunct to papilledema evaluation to diagnose potentially elevated ICP [21].

This study compares the diagnostic accuracy of US-ONSD, US-ODE, and fundoscopic papilledema in children with symptoms compatible with raised ICP to detect intracranial pathology with treatment indication.

In addition, we investigated the repeatability and reliability of US-ODE measurement and sought to define US-ONSD and US-ODE cut-off values associated with potentially elevated ICP and the presence of fundoscopic papilledema.

## Methods

### Study design

Paediatric patients were included in this prospective observational study (2018–2021) according to the following criteria: All patients presented with clinical symptoms suggestive for raised ICP (headache, vomiting, irritability, visual disturbance, e.g. blurred/double vision). Some of them appeared for the first time, some were already known and pre-treated, and all required further investigation due to the duration or severity of symptoms. All patients underwent fundoscopy by independent ophthalmologists (inpatient/outpatient). Additionally, all patients underwent transorbital ultrasound of ONSD and ODE by an investigator blinded to fundoscopy. US findings were not included in decision making regarding further diagnostics and treatment. Only patients tolerating US investigations without sedation were included. The maximum time-interval between fundoscopy and transorbital US was 24 h, with no ICP-affecting therapy in between. Depending on kind, duration and severity of symptoms and fundoscopic findings, further imaging diagnostics were performed.

In 28 patients, an additional invasive ICP measurement (by intraparenchymal ICP probe) was performed, either because the diagnostic work-up required it, e.g. in PTC [22], or the preceding diagnostics did not yield clear findings (e.g. arachnoid cyst or clinical suspicion of craniostenosis in absence of papilledema).

According to findings and treatment decisions, patients were stratified into 2 groups: treatment group (TG) and non-treatment group (NTG).

Inclusion criteria for TG included the following in addition to the existing symptoms as described above: (1) imaging evidence of disease/disease-recurrence associated with increased ICP (hydrocephalus, space-occupying arachnoid cyst, brain tumour, craniosynostosis with copper-beaten skull or involvement of multiple sutures) and/or (2) evidence of raised intracranial pressure ≥ 18.5 mmHg (≥ 20.5 mmHg if child was sedated and/or obese) and/or (3) diagnosis of PTC according to the Friedman criteria [22].

Inclusion criteria for NTG encompassed the following: (1) absence of fundoscopic papilledema and absence of radiological evidence of a disease associated with increased ICP or (2) absence of fundoscopic papilledema, absence of imaging findings and intracranial pressure < 18.5 mmHg.

Informed consent was obtained from parents and children old enough to understand the study prior to US-investigation. According to the written ophthalmologic reports, fundoscopic findings were classified into (A) papilledema (in any manifestation) and (B) no papilledema.

All procedures were in accordance with the Code of Ethics of the World Medical Association (Declaration of Helsinki). The study protocol was approved by the institutional ethics committee (180/2018BO2). The study meets the STROBE guidelines for reporting observational studies.

### Study population

Seventy-two patients, aged 9 months to 18 years, (mean age 9.3 ± 4.7 years), were included; 69 children were > 1 year. Forty-six children were male (64%), and 26 were female (36%).

The diagnoses included: PTC (*n* = 21), brain tumor (*n* = 16), arachnoid cyst (*n* = 8), craniosynostosis (*n* = 9), other intracranial pathologies (sinus vein thrombosis, intracranial haemorrhage, hydrocephalus; *n* = 14) and patients without proven disease (*n* = 4).

In 40/50 patients of the TG, fundoscopy, US-ONSD and US-ODE were acquired before and after ICP decreasing therapy. In 24 of those 40 patients, at least one further follow-up investigation was performed (maximum follow-up interval 50 weeks).

### PoC ultrasound

One examiner (SRK), highly experienced in transorbital ultrasound, performed all US-investigations. US was performed in supine position, head straight and not elevated. A 12-MHz linear transducer (Philips, Epiq 5G) was placed to the closed eyelid. US-ONSD determination was performed 3 mm behind the bulb, 90° to the optic nerve in axial plane (Fig. 4e). From three measurements per eye, mean ONSD and resulting mean binocular US-ONSD were calculated as previously described [23]. Additionally, the optic disc elevation was measured three times per side (Fig. 4e) and mean binocular US-ODE was calculated.

### Sample size calculation

The sample size estimation for comparison of US-ONSD means was calculated at 16 patients per group to achieve 99% power with an alpha of 0.1 (*p* < 0.001). The sample size estimate for comparison of US-ODE means was calculated at 22 patients per group to achieve 95% power with an alpha of 1 (*p* < 0.01). With an estimated loss of 10% due to exclusion criteria and data loss, the study aimed to recruit a minimum of 50 patients.

### Statistical analysis

SPSS (PASW Statistics 27, IBM) statistical software was used. Data were tested for normal distribution using the Kolmogorov-Smirnov or Shapiro-Wilk test. Parametric data were reported as means $\pm$ standard deviation (sd). Depending on normality of distribution, correlation of parametric variables was tested using Pearson’s or Spearman’s correlation; point-biserial correlation *r* was used for comparing parametric to nominal variables. Receiver operating characteristic (ROC) curves were created to define US-ONSD and US-ODE cut-off values according to Youden-Index calculation. Analysis of variance (ANOVA) was used to determine intra-observer variability of US-ODE measurement. Cronbach’s alpha (α), a model of internal consistency, was used to test repeatability and observer reliability of US-ODE determination. Repeatability and reliability are considered reasonable when Cronbach’s alpha is > 0.7, strong if > 0.8 and excellent if > 0.9. The Mann-Whitney *U* test and dependent *t* test were used for comparing mean values or paired samples, respectively. Chi-Quadrat-test was applied for comparing qualitative parameters. Bonferroni’s correction was used to correct multiple comparisons. Statistical significance was set at *p* < 0.05: *p* < 0.05*, *p* < 0.01**, *p* < 0.001***.

## Results

In 50/72 children, the subsequent diagnostic procedures (see under “Study design” Section) resulted in primary diagnosis or determination of recurrence of intracranial disease requiring treatment (TG). Diagnoses encompassed PTC (19/50), space-occupying brain tumours or tumours with hydrocephalus (13/50), craniostenosis (6/50), space-occupying arachnoid cysts (7/50) and hydrocephalus of different origin (5/50). Treatment depended on the underlying diagnosis, like lumbar CSF drainage, tumour-resection, endoscopic cyst fenestration, expansion cranioplasty, VP-Shunt (revision) or acetazolamide medication.

Four of 22 remaining patients presented for the first time, 18 were already known for previously treated PTC (2/22), brain tumour (3/22), craniosynostosis (3/22), arachnoid cyst (1/22) and other neuropaediatric diseases (see “Study population” Section) (9/22). According to definitions above (“Study design” Section), intracranial disease with increased ICP requiring treatment was ruled out in the four previously unknown children and recurrence in the 18 other children (non-treatment group = NTG).

### Repeatability and intra-observer reliability of US-ODE measurement

Repeatability of US-ODE measurements was excellent with Cronbach’s *α* = 0.978 (left) and 0.989 (right). A scan-rescan reproducibility analysis of two US-ODE measures on each side yielded a mean bias of 0.006 mm (range − 0.24 to 0.4 mm) and 0.001 mm (range − 0.3 to 0.2 mm) left and right side, respectively.

To test for intra-observer variability, sequential measurements of US-ODE were performed in 20 random patients at two different timepoints, without therapy affecting ICP in between. The interval between investigations was 5 days to 6 months. The examiner was blinded to results obtained at the first timepoint. Mean US-ODE was 0.22 ± 0.33 mm at first and 0.19 ± 0.32 mm at second investigation. Testing for intra-observer reliability demonstrated eminent correlation (Cronbach’s *α* = 0.985).

### Initial US-ONSD, US-ODE and fundoscopy in relation to treatment indication

**Fig. 1:**
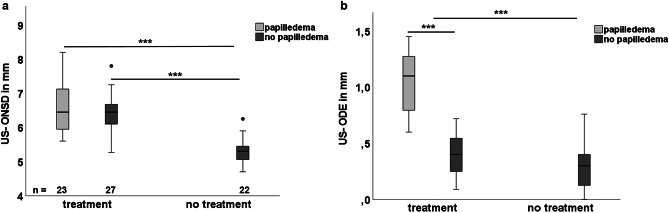
Initial US-ONSD and US-ODE in symptomatic patients considering fundoscopic ODE findings and indication for treatment. **a** US-ONSD and **b** US-ODE at initial presentation of 72 children with signs and symptoms of raised ICP. ***p < 0.001

Twenty-three of 50 patients in TG (46%) had papilledema at fundoscopy, in contrary to 0/22 in NTG (Fig. 1).

Mean US-ONSD in TG was 6.5 ± 0.6 mm compared to 5.31 ± 0.38 mm in NTG (mean ΔUS-ONSD 1.19 mm, *p* < 0.001). TG patients with papilledema had insignificant larger US-ONSD values (6.59 ± 0.74 mm) compared to those without (6.43 ± 0.53 mm).

The mean US-ODE in TG was 0.7 ± 0.4 mm compared to 0.31 ± 0.2 mm in NTG (ΔUS-ODE 0.39 mm, *p* < 0.001). In TG patients with and without papilledema, the mean US-ODE was 1.06 ± 0.27 mm and 0.40 ± 0.19 mm, respectively (*p* < 0.001). In patients without papilledema, the US-ODE difference between TG (0.40 ± 0.19 mm) and NTG (0.31 ± 0.22 mm) was not significant (Fig. 1b).

### Correlation of US-ONSD, US-ODE, fundoscopic papilledema and ICP

US-ONSD correlated poor to both fundoscopic papilledema (*r* = 0.397**) and US-ODE (*r* = 0.496**).

The correlation of US-ODE and fundoscopic papilledema was excellent (*n* = 72, *r* = 0.821***).

Of 28 patients with invasive ICP measurement, 23 had raised ICP, 7/23 showed fundoscopic papilledema. Mean ICP of children with papilledema was 35.8 ± 10.4 mmHg compared to 29.3 ± 5.5 mmHg in those without (*p* > 0.05).

**Fig. 2:**
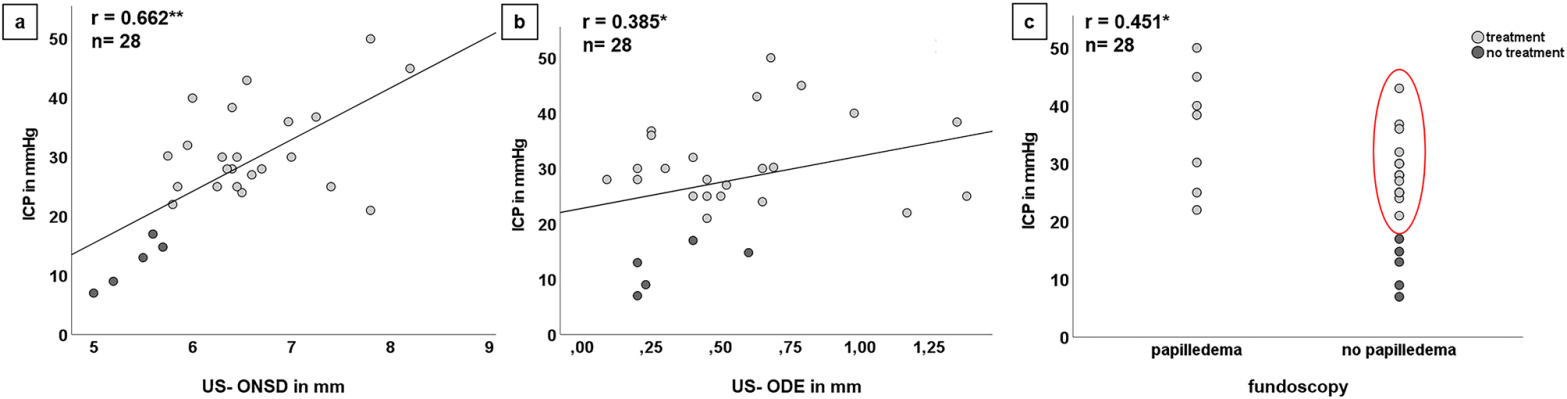
Correlation of invasive ICP values with US ONSD (**a**), US ODE (**b**) and fundoscopic ODE findings (**c**) in 28 children considering indication for treatment. Grey dots correspond to children from TG, black dots from NTG. In graph **c**, the red circle marks children with indication for treatment and abnormal ICP values (20–45 mmHg) without papilledema

US-ONSD revealed the best correlation to ICP (*r* = 0.662**). Correlation of ICP to US-ODE (*r* = 0.385*) and to fundoscopic papilledema (*r* = 0.451*) was poor (Fig. 2).

### US-ODE cut-off value to detect papilledema by fundoscopy

**Fig. 3:**
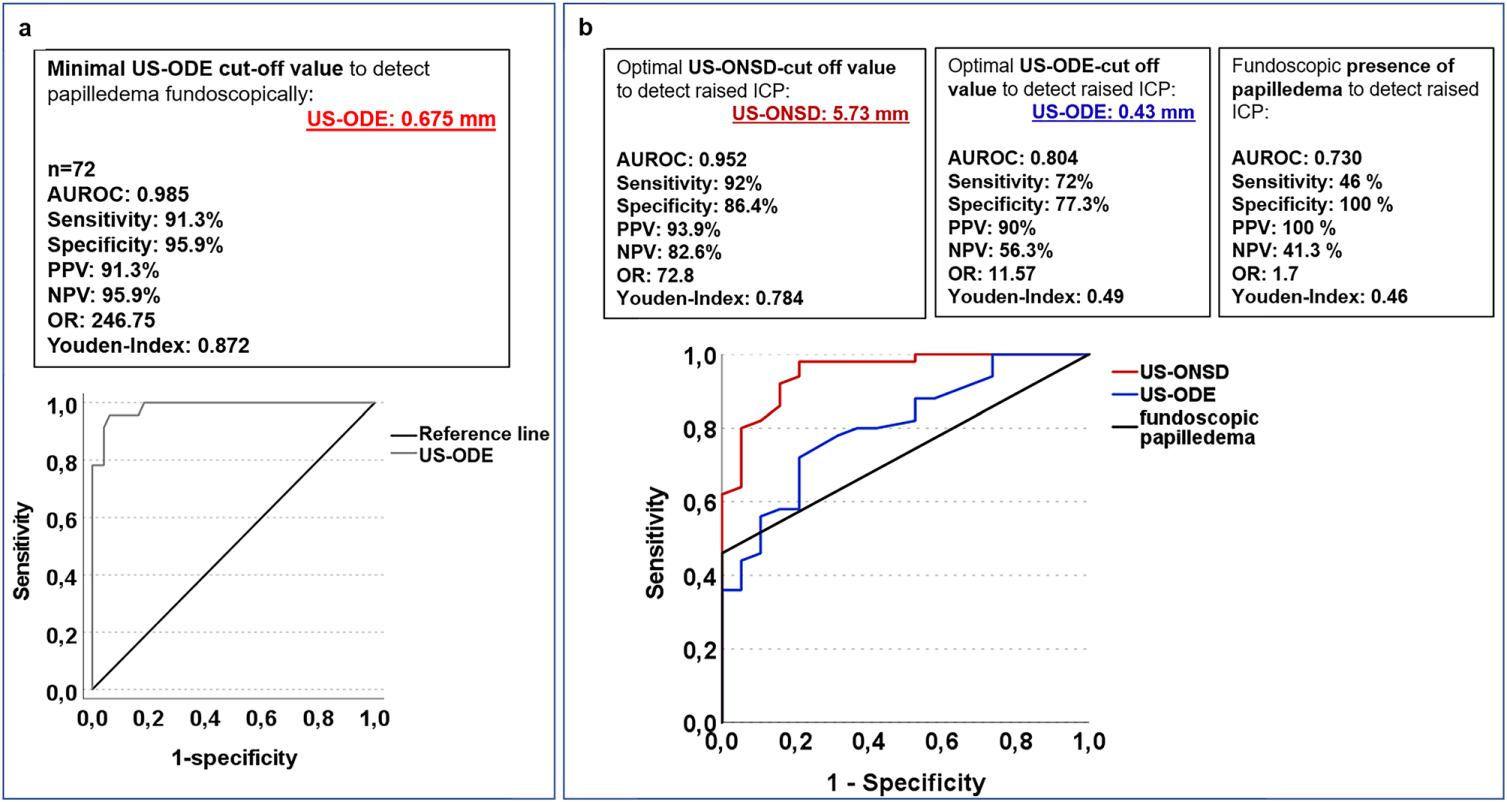
ROC analysis of fundoscopic findings, US-ONSD and US-ODE. **a** AUROC curve for minimal US-ODE cut-off value to detect papilledema fundoscopically. **b** AUROC curve of US-ONSD and US-ODE cut-off value to detect raised ICP requiring treatment in children, compared to AUROC curve of fundoscopic papilledema. PPV, positive predictive value; NPV, negative predictive value; OR, odds ratio; AUROC, area under the receiver operating characteristic curve

We sought to determine the minimal US-ODE that manifests as fundoscopic papilledema. ROC analysis identified 0.675 mm as cut-off value to detect papilledema by fundoscopy with excellent diagnostic accuracy (Fig. 3a).

### ROC analysis of US-ONSD, fundoscopic papilledema and US-ODE to detect potentially raised ICP

**Fig. 4:**
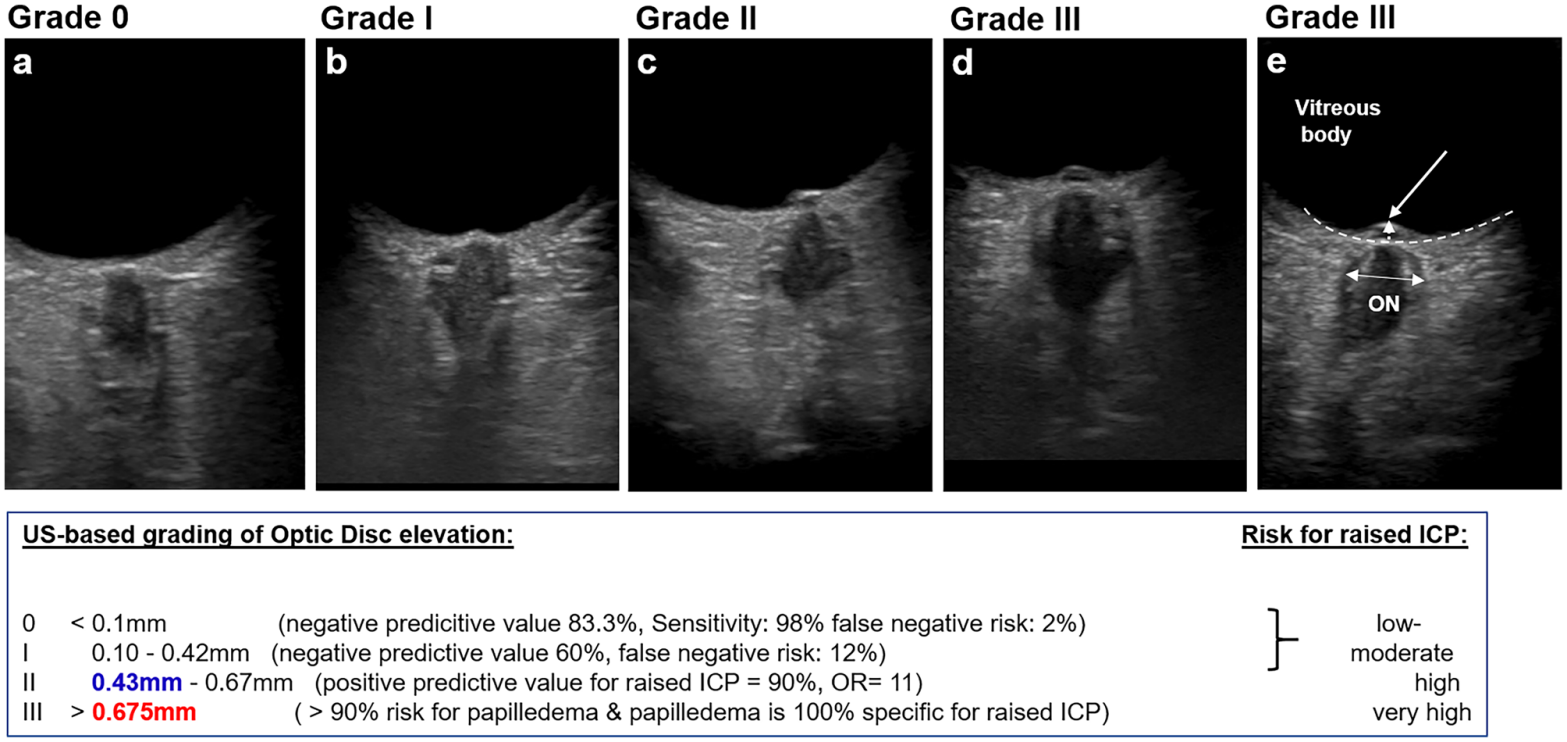
Ultrasound-based grading of optic disc elevation (ODE). **a** Grade 0 (mean US-ODE 0.087 mm) in patient without papilledema and normal ONSD, no pathology. **b** Grade I (mean US-ODE 0.23 mm), no papilledema, small ONSD value, no pathology. **c** Grade II (US-ODE 0.52 mm), no papilledema, but pathology requiring treatment and ONSD enlargement. **d**, **e** Grade III (US-ODE 1.21 mm), fundoscopic papilledema, large US-ONSD and diagnosis with need for treatment. **e** US-based ONSD and ODE measurement. Dotted line marks retina. White double-headed arrow marks area of ONSD measurement. Optic disc elevation is measured from retina at the entrance of the optic nerve (ON) to the maximum of elevation (white arrows) within the vitreous body. **a**–**e** show transorbital, B-scan-ultrasonographic images

The optimal US-ONSD cut-off value to detect potentially raised ICP was 5.73 mm with excellent diagnostic accuracy (Fig. 3b). Fundoscopic papilledema showed a specificity of 100% to detect potentially raised ICP, with a sensitivity of 46%. The ideal US-ODE cut-off value was 0.43 mm with good diagnostic accuracy, better than papilledema on fundoscopy but inferior to US-ONSD. The combination of US-ONSD and US-ODE (AUROC: 0.745, sensitivity: 70%, specificity: 78.9%) could not increase diagnostic accuracy compared to US-ODE alone. Based on the results from “US-ODE cut-off value to detect papilledema by fundoscopy” and “ROC-analysis of US-ONSD, fundoscopic papilledema and US-ODE to detect potentially raised ICP” Sections, a US-based grading system of optic disc elevation was established (Fig. 4).

### US-ONSD, US-ODE and fundoscopic papilledema after therapy

**Fig. 5:**
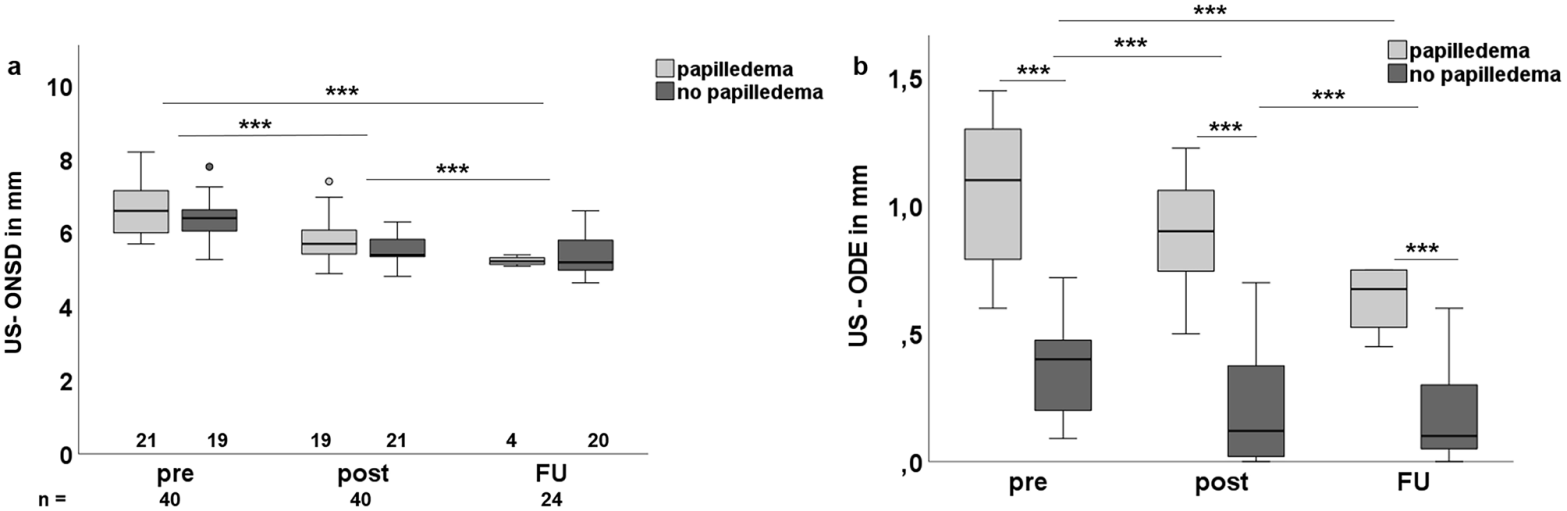
US-ONSD, US-ODE and fundoscopic findings before and after therapy. **a** US-ONSD and **b** US-ODE considering fundoscopic findings before and after therapy and during long-term-follow-up. ***p < 0.001

All 50 patients benefited from therapy; relapse was ruled out clinically, radiologically and/or invasively. Forty of 50 patients underwent fundoscopy, US-ONSD and US-ODE before and within 1–14 days after therapy (Fig. 5). In 24/40 children, US investigations were repeated further (2–50 weeks). At initial examination, 21/40 patients (52.5%) had papilledema. Initial mean US-ONSD was 6.53 ± 0.66 mm with no difference between patients with/without papilledema (6.64 ± 0.7 mm vs. 6.40 ± 0.55 mm). Initial mean US-ODE was 0.73 ± 0.42 mm with significant difference between patients with/without papilledema (1.06 ± 0.27 mm vs. 0.37 ± 0.19 mm, *p* < 0.001).

After therapy, 19/21 children still had papilledema; however, US-ONSD decreased significantly to 5.74 ± 0.54 mm without difference between those with (5.83 ± 0.64 mm) and without papilledema (5.59 ± 0.38 mm). Overall US-ODE decreased significantly to 0.53 ± 0.4 mm (*p* < 0.001) with a significant difference between those with and without papilledema (0.89 ± 0.22 mm vs 0.22 ± 0.22 mm, *p* < 0.001).

At end of follow-up, four patients had persistent papilledema (2 patients each with initial tumour and PTC; time intervals were 2, 8, 12, and 24 weeks after therapy). However, US-ONSD significantly decreased to normal values (5.29 ± 0.4 mm) in all. US-ODE significantly decreased as well (0.28 ± 0.25 mm, *p* < 0.001), but the four children with persistent papilledema had significantly elevated US-ODE (0.64 ± 0.14 mm) compared to those without (0.19 ± 0.2 mm).

## Discussion

This study is the first to compare the diagnostic validity of US-ONSD, US-ODE, and fundoscopic papilledema to detect raised ICP with treatment indication in children. It is the largest prospective paediatric cohort used to define US-ONSD and US-ODE cut-off values to identify elevated ICP in need of treatment. Regression dynamics for all parameters after therapy were analysed. Finally, a US-based classification of the optic disc elevation as additional first-line screening tool for raised ICP in children was developed.

### US-ONSD is an excellent non-invasive screening tool for detecting potentially elevated ICP requiring treatment

In children, symptoms like headache and vomiting are common and unspecific [24] but can indicate a significant ICP increase. The standard non-invasive, diagnostic tool is fundoscopy. The misconception that absence of papilledema is diagnostic to rule out raised ICP is still very common [25], although it is known that increased ICP may occur without papilledema in 40–60% of cases [8, 11–13]. In an infant study, only 1/56 children with raised ICP had fundoscopic papilledema [10]. Part of the pathophysiology of papilledema is the translation of ICP to the peri-optic subarachnoid space inside the dural optic nerve sheath (ONS) [26]. A pressure rise within the ONS results in a widening of the elastic ONS and increase of ONSD. The response time of the ONS to pressure changes is prompt due to the elasticity-coefficient of the dura [27]. As one result of persistently raised pressure within the ONS, an optic disc swelling can occur as a secondary late event [28]. US-ONSD, which measures the primary event in the pathophysiological chain that may lead to papilledema, is a reliable tool for assessing ICP in children with excellent intra-individual correlation of ONSD and ICP and a rapid decline in ONSD after ICP reduction [29]. Furthermore, in presence of optic nerve atrophy, papilledema is unlikely to occur at all, whereas an increase of ONSD will still occur in response to raising ICP [28].

The use of ONSD measured via US or MRI/CT [30] has been described in children [31–33], but a prospective comparison between ONSD, ODE and papilledema in symptomatic patients with/without potentially elevated ICP has not yet been made.

We demonstrate that children with confirmed underlying diseases associated to increased ICP had significant higher US-ONSD values (mean 6.5 ± 0.63 mm) compared to those without disease (mean 5.31 ± 0.38 mm, comparable to reference ONSD cut-off values of 5.2/5.3 mm for ICP < 10 mmHg [17, 18]). Most importantly, and in agreement with literature [34], more than 50% of the patients of the TG had no fundoscopic papilledema. These patients had — accordingly — significantly lower US-ODE values.

A correlation between US-ODE and fundoscopic papilledema is existent, and a highly diagnostic cut-off value for US-ODE was identified above which papilledema was found fundoscopically. The correlation analysis between US-ONSD, US-ODE and papilledema with invasively measured ICP showed convincing results for US-ONSD, but not for US-ODE or fundoscopic papilledema. Figure 2 shows that individual children with ICP values between 25 and 45 mmHg had no papilledema and would have been missed using fundoscopy as only screening tool. The ROC analysis revealed that the diagnostic accuracy of an US-ONSD cut-off value of 5.73 mm is clearly superior compared to the use of US-ODE or fundoscopy. This US-ONSD cut-off value is concurrent to a previously published cut-off value of 5.75 mm to detect ICP > 20 mmHg in children [17, 18].

### US-ONSD is an outstanding tool to monitor treatment outcome

Confirmation of success after treatment of elevated ICP is of clinical importance. Several studies described a relevant reduction of ONSD after treatment [29, 31, 35], and an immediate ONSD decrease after ICP lowering is known [29]. The mean time-interval for papilledema development is 7–10 days [36], indicating that an acute ICP elevation is not rapidly followed by papilledema. The resolution time of papilledema is even longer with 6–10 weeks [28, 37, 38].

In our cohort, elevated US-ONSD values decreased significantly after therapy, but were still above normal 1–14 days after therapy. Of this cohort, 47.5% had no initial papilledema. Papilledema persisted in 19/21 patients at first investigation after therapy. After a follow-up of 2–50 weeks, US-ONSD values normalized (5.29 ± 0.4 mm), whereas 4 patients had persistent papilledema (2–24 weeks after therapy). Most importantly, US-ONSD values did not differ between patients with/without papilledema. These results suggest that in contrary to papilledema, treatment success is immediately reflected by an ONSD decrease. The regression of elevated ONSD values shows a hysteresis effect, since a longer-lasting ONS distention leads to a (transient) loss of dural elasticity in terms of a memory effect, which precludes a fast return to normal ONSD values. Hysteresis probably depends on extent and duration of ICP increase [39] and type of pathology [29].

In summary, US-ONSD can, unlike papilledema, reliably demonstrate a treatment success in very early stages, and it can be quantified in numbers.

### ODE can be measured easily and reliably with US

A few recent studies reported on US-ODE examination as a tool to detect papilledema and increased ICP in adults [40–42] and children [20]. Tessaro et al. published a retrospective pilot-study on 40 children and compared US-ODE and US-ONSD values with invasively measured ICP [43]. Twenty-six children had elevated ICP, and the optimal ODE cut-off to detect raised ICP was 0.66 mm (sensitivity-96%, specificity-93%). In our study indications for treatment were not only based on invasively measured ICP values but also on clear radiologic findings such as massive hydrocephalus, space-occupying tumours or secondary craniostenosis with massive copper beaten skull appearance in X-ray, known to result in elevated ICP [44, 45]. We found the same US-ODE cut-off value of 0.675 mm associated to papilledema, suggesting that a relevant ICP increase seems to manifest on fundoscopy only if ODE exceeds 0.7 mm.

However, the US-ODE cut-off value with best diagnostic accuracy to detect potentially raised ICP requiring treatment was lower at 0.43 mm with a PPV of 90%. This suggests that patients with an US-ODE grade II (0.43–0.67 mm) are very likely to carry a significantly raised ICP, however will not be detected by fundoscopy. The diagnostic sensitivity of fundoscopic papilledema is much lower and inferior to US-ODE using our grading system. US-ODE values are furthermore quantitative numbers, which can reveal even smaller differences and thus carry more information than purely qualitative fundoscopic findings. US-ODE showed a high repeatability and intra-observer reliability, similar to another recent study that additionally demonstrated an excellent interrater reliability [46].

### Limitations

The major limitation of this study is, despite its prospective nature, the relatively small cohort especially in the subgroups. Another limitation is that fundoscopic findings were dichotomized only (papilledema vs. no papilledema) without consideration of the papilledema severity. However, in clinical routine, especially at initial presentation, the relevant and often only message in the written report is “presence or absence” of papilledema, due to the high specificity of papilledema. The Frisén-rating of severity of papilledema [47, 48] is very much examiner dependent and its reproducibility and discriminative ability is limited even among ophthalmologists [49].

## Conclusions

US-ONSD and US-ODE are a reliable, non-invasive, diagnostic method for detecting potentially elevated ICP superior to fundoscopy. In addition, US-ONSD and US-ODE can indicate treatment success earlier and more reliably. US-ONSD and US-ODE cut-off values can be used in screening for potential and treatment-relevant ICP elevation in children. The proposed US-based grading scale of ODE is a further adjunct to support diagnosis.

## Data Availability

The datasets generated during and/or analyzed during the current study are available from the corresponding author on reasonable request.
